# Longitudinal change in neurocognitive functioning in children and adolescents at clinical high risk for psychosis: a systematic review

**DOI:** 10.1007/s00787-023-02221-9

**Published:** 2023-05-18

**Authors:** Borja Pedruzo, Claudia Aymerich, Malein Pacho, Jon Herrero, María Laborda, Marta Bordenave, Anthony J. Giuliano, Robert A. McCutcheon, Luis Gutiérrez-Rojas, Philip McGuire, William S. Stone, Paolo Fusar-Poli, Miguel Ángel González-Torres, Ana Catalan

**Affiliations:** 1 Department of Psychiatry, Basurto University Hospital, Bilbao, Spain; 2 Worcester Recovery Center and Hospital, Massachusetts Department of Mental Health, Boston, USA; 3 Department of Psychosis Studies, Institute of Psychiatry, Psychology and Neuroscience, King’s College London, London, UK; 4 Department of Psychiatry, University of Oxford, Oxford, UK; 5 Psychiatry Service, San Cecilio University Hospital, Granada, Spain; 6 National Institute for Health Research Biomedical Research Centre, London, UK; 7 Outreach and Support in South London Service, South London and Maudsley National Health Service Foundation Trust, London, UK; 8 Department of Psychiatry, Beth Israel Deaconess Medical Center, Harvard Medical School, Boston, MA USA; 9 Early Psychosis: Interventions and Clinical-Detection (EPIC) Lab, Department of Psychosis Studies, Institute of Psychiatry, Psychology and Neuroscience, King’s College London, London, UK; 10 Department of Brain and Behavioral Sciences, University of Pavia, Pavia, Italy; 11 Neuroscience Department, University of Basque Country (UPV/EHU), Leioa, Spain; 12 Biocruces Bizkaia Health Research Institute, Barakaldo, Spain; 13 CIBERSAM. Centro Investigación Biomédica en Red de Salud Mental, Madrid, Spain

**Keywords:** Neurocognition, Clinical high risk, Child, Adolescent, Psychosis

## Abstract

Clinical high risk of psychosis (CHR-P) population has become an attractive area of interest in preventing transitions to psychosis. The consequences of developing a psychotic disorder may be worse in cases of early onset. Thus, childhood and adolescence represent a critical developmental window, where opportunities to gain social and adaptive abilities depend on the individuals’ neurocognitive performance. There have been previous syntheses of the evidence regarding neurocognitive functioning in CHR-P individuals and its longitudinal changes. However, there has been less focus on children and adolescents at CHR-P. A multistep literature search was performed from database inception until July 15th, 2022. PRIMSA/MOOSE compliant systematic review and PROSPERO protocol were used to identify studies reporting on longitudinal changes in neurocognitive functioning in children and adolescents (mean age of sample ≤ 18 years) at CHR-P and matched healthy control (HC) group. A systematic review of identified studies was then undertaken. Three articles were included, resulting in a total sample size of 151 CHR-P patients [mean (SD) age, 16.48 (2.41) years; 32.45% female] and 64 HC individuals [mean (SD) age, 16.79 (2.38) years; 42.18% female]. CHR-P individuals had worse outcomes in verbal learning, sustained attention and executive functioning domains compared to HC. Individuals taking antidepressants had better outcomes in verbal learning in contrast with those taking antipsychotics. In children and adolescents, neurocognition may be already impaired before the psychosis onset, and remains stable during the transition to psychosis. Further study should be performed to obtain more robust evidence.

**Supplementary Information:**

The online version contains supplementary material available at 10.1007/s00787-023-02221-9.

## Introduction

Diagnosis and treatment of psychotic disorders typically occur following the onset of a first episode of psychosis. Despite the current discrepancies regarding potential risk factors or prognosis in psychotic disorders, different investigation lines agree that childhood and adolescence are a window of opportunity for prevention [1, 2] and early intervention [3–7]. Indeed, in the last two decades, clinical high-risk stages of psychosis, which frequently occur in those aged under 18 years [8], have become an increasingly attractive area of interest in an attempt to prevent potential transitions to psychosis [9, 10].

In this context, specific criteria have been developed to identify individuals with a prospective clinical high risk for psychosis (CHR-P) [11]. Currently, CHR-P states are clinically segregated into three subgroups: attenuated psychotic symptoms (APS), brief limited intermittent psychotic symptoms (BLIPS), and genetic risk and functioning deterioration syndrome (GRFD). Of these, the APS subtype represents a wide majority (85%) of all the CHR-P states [9].

The deleterious consequences of developing a psychotic disorder may be aggravated when it has an early onset (EOP), i.e., before age 18 [12]. Compared with adult-onset psychosis, individuals with EOP are more likely to possess adverse prognostic criteria, such as more negative symptoms at the onset [12], lower premorbid adjustment [13], and more substantial neurodevelopmental deficits [14]. Current literature states contradictory findings in this population: whereas some studies suggest that people with EOP have worse clinical and functional outcomes [15], some studies have reported similar short-term and better long-term outcomes of EOP compared to adult-onset psychosis [16]. Transition risk to psychosis of CHR-P individuals varies over time, cumulating to 0.20 (95% CI 0.19–0.21; *n* = 2357) at 2 years and 0.35 (95% CI 0.32–0.38; *n* = 114) at 10 years [17]. However, most who will not develop psychosis still present substantial mental health burdens at follow-up [10, 18]. The prognostic accuracy of CHR-P instrument is excellent, although this is only based on group-level prognostications [19]. The prevalence of CHR-P features is about ten times higher in clinical samples (19.2%) than in the general population (1.7%). The combination of the relatively high risk of developing psychosis with a low prevalence in the population yield an associated global population attributable fraction of about 10% [20]. Neurocognitive impairment is a core feature of psychosis [21] and may be used as a biomarker to identify individuals at CHR-P and adjust their risk of transitioning to psychosis [22].

Childhood and adolescence represent a critical developmental window, where opportunities to gain social and adaptive abilities depend on the individuals' neurocognitive performance, especially in the early stages of psychosis [23]. Many existing syntheses exist regarding current knowledge about neurocognitive functioning in CHR-P individuals [22–24], and specifically about longitudinal changes across time in this population [25]. However, the empirical literature on the neurocognitive performance of children and adolescents at CHR-P is much less extensive, and there is a need for an updated review [26–30]. The main aim of this study is to provide a systematic review and meta-analytical examination of longitudinal neurocognitive outcomes in children and adolescent population at CHR-P.

## Methods

The study protocol was registered on PROSPERO (CRD42022352291) and was conducted following the Preferred Reporting Items for Systematic Reviews and Meta-Analyses reporting guideline [31] (PRISMA, Table S1 in the Supplement), the Meta-analysis of Observational Studies in Epidemiology (MOOSE) reporting guideline [32] (MOOSE, Table S2 in the Supplement) as long as results allow a meta-analytic approach, and the Enhancing the Quality and Transparency of Health Research (EQUATOR) reporting guideline [33].

### Search strategy and selection criteria

A systematic, multistep literature search (search terms appended in Methods S1 in the Supplement) was implemented by 2 independent researchers (B.P., C.A.). Web of Science database (Clarivate Analytics), incorporating the Web of Science Core Collection, BIOSIS Citation Index, KCI-Korean Journal Database, MEDLINE, Russian Science Citation Index, and SciELO Citation Index, as well as Cochrane Central Register of Reviews, PubMed, and Ovid/PsycINFO databases were searched until July 15th, 2022. Abstracts of articles identified were screened and, after excluding those not relevant, the full texts were assessed for eligibility. The references of previously published meta-analyses and systematic reviews and of the articles included were then manually searched.

Studies were included if they (1) were primary studies enrolling individuals with clinical high risk for psychosis, defined according to validated CHR-P psychometric interviews (Methods S2 in the Supplement); (2) reported neurocognitive tasks (Methods 3 in the Supplement); (3) reported longitudinal changes in neurocognition from the time of the onset of the disorder over follow-up; (4) included a control group, preferably HC, or stratified neurocognitive functioning according to longitudinal transition to psychosis (5) included population with mean age ≤ 18; (6) were written in English. Studies were excluded if they (1) were reviews, clinical cases, abstracts, conference proceedings, or study protocols; (2) were studies using a non-established definition of clinical high risk for psychosis; (3) used non-established CHR-P psychometric interviews (Methods S2 in the Supplement); (4) lacked an HC group or data stratification on the transition to psychosis, and (5) were studies in languages other than English. When there were two or more overlapping studies, the largest sample of CHR-P was chosen.

### Outcome measures and data extraction

Four researchers (M.P., J.H., M.L., M.B.) independently extracted data from all identified studies (Methods S4 in the Supplement). The databases were then cross-checked and discrepancies were resolved through consensus under the supervision of a senior researcher (A.C.). Neurocognitive tasks were clustered into 7 Measurement and Treatment Research to Improve Cognition in Schizophrenia (MATRICS) domains [34, 35], namely, (1) processing speed, (2) attention or vigilance, (3) working memory, (4) verbal learning, (5) visual learning, (6) reasoning and problem-solving, and (7) social cognition (Methods S3 in the Supplement). To ensure the comprehensiveness of our review, we also considered additional CHR-P tasks that had been included in studies of this population and that are not included in the more limited MATRICS framework (Methods S3 in the Supplement). These tasks were categorized by some senior experts (A.G., W.S.) into the following eight domains: (1) general intelligence, (2) premorbid IQ, (3) visuospatial ability, (4) verbal memory, (5) visual memory, (6) executive functioning, (7) motor functioning, and (8) olfaction.

Notwithstanding the aim to conduct a meta-analysis to synthesize the available evidence on longitudinal neurocognitive changes in children and adolescents at CHR-P, after a comprehensive search, a screening process and data extraction, it was found that the data available were not sufficient to allow for a quantitative analysis.

### Systematic review

As a result of the insufficient data for a meta-analysis, only a systematic review was performed to provide a comprehensive overview of the existing literature on the present topic. The systematic review was performed according to the PRISMA [31] guidelines. The quality of the studies was assessed using the Newcastle–Ottawa Scale (NOS) [36]. Any disagreements were resolved through discussion and consensus.

## Results

**Fig. 1:**
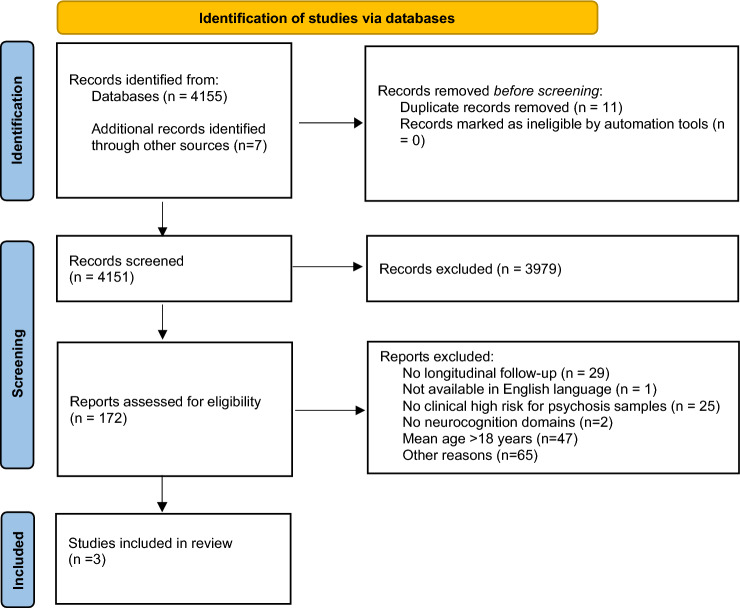
PRISMA 2020 flow diagram outlining the study selection process

As described in Fig. 1, the literature search yielded 4155 citations through electronic database. In addition, 7 citations where identified through other sources, given a total of 4162 citations screened for eligibility. 172 articles were assessed in full text, and 169 were excluded, being the mean age of the samples the principal reason for exclusion. The final database for the systematic review included 3 studies, as it can be seen in Fig. 1. Due to the limited number of studies that were finally included and the wide variety of neurocognitive domains and tasks, there was not enough data to be quantitative meta-analyzed. Therefore, only a systematic review was undertaken. Characteristics of the included studies are described in Table 1.

**Table 1:** Characteristics of included studies

References	Country	N CHR-P	N HC	Age mean (SD)	FU (months)	Task used	NOS	Results summary
Bowie et al. [37]	Canada	53	17	16.21 (2.01)	6	CVLT total learning; COWAT; TMT-A; TMT-B; Letter-number Span (MATRICS); CPT shapes; CPT digits	7	Learning and verbal memory: worse functioning at BL in CHR-P groups. Improvement in CVLT Total Learning in AD group compared to AP group
								Sustained attention: improvement in the AD and medication-naïve groups
								Verbal fluency: stable COWAT scores between BL and FU in CHR-P
								No correlation between neurocognitive changes and psychopathology
Woodberry et al. [39]	USA	53	32	16.11 (2.48)	12	Wide Range Achievement Test (WRAT-3) Reading (Blue); Wechsler Abbreviated Scale for Intelligence (WASI): vocabulary, similarities, block design, matrix reasoning; 2 test IQ estimate (WASI vocabulary and WASI matrix reasoning);CPT-IP-II four digits; CPT-IP-II shapes; CVLT-II (> 16) or CVLT Child Version (< 16); WMS-III Logical Memory (> 16) or CMS (< 16); LNS; D-KEFS; Verbal Fluency Condition 3 and Trail Making Condition 4; WCST-128: Computer Version; WMS-III (≥ 16) or WISC-IV (< 16) letter-number sequencing; Finger Tapping Test; B-SIT	7	Learning and verbal memory: stable scores in verbal memory in the HC relative to CHR-P
								Sustained attention: no difference between HC and CHR-P groups
								Verbal fluency: stable COWAT scores between BL and FU in CHR-P
								No bigger overall neurocognitive impairment at FU for CHR-P who transitioned
Carrion et al. [38]	USA	45	15	17.6 (1.73)	Mean (SD): 8.04 (10.26) after psychosis onset	WAIS-R/WISC-III; CVLT Total for trials 1–5 and Long delay free recall; Digit span forward and backward; Letter-number span; WSCT Perseverative errors, Categories completed and Conceptual level responses; COWAT; CPT-IP; TMT-A; TMT-B; WISC-III/WAIS-R digit symbol coding; WRAT-III Reading; WAIS-R/WISC-III vocabulary	7	Learning and verbal memory: stable scores in verbal memory in the HC relative to CHR-P
								Processing speed: impairment at BL in CHR-P patients persisted after psychosis onset
								CHR-P that transitioned had bigger impairment in global neurocognitive and intellectual perf. from BL to FU, with no deterioration after psychosis onset
								Worsening of positive symptoms was associated to improvements in processing speed, sustained attention, working memory, and global cognition. Improvement in working memory and global cognition were related to more negative symptoms and disorganized symptoms, respectively

AD, antidepressant; AP, antipsychotic; BL, Baseline; BSIT, Brief Smell Identification Test; CHR-P, Clinical High Risk for Psychosis; CMS, Children's Memory Scale Stories; COWAT, Controlled Oral Word Association Test; CPT, Continuous Performance Test; CPT-IP-II, Continuous Performance Test-Identical Pairs; CVLT, California Verbal Learning Test; D-KEFS, Delis–Kaplan Executive Function System; EF, Effect-size FU, Follow-up; HC, healthy control; IQ, Intelligence Quotient; LNS, Letter–Number Span; NOS, Newcastle–Ottawa Scale; SD, Standard Deviation; TMT-A, Trail Making Test-Part A; TMT-B, Trail Making Test-Part B; WAIS, Wechsler Adult Intelligence Scale; WASI, Wechsler Abbreviated Scale of Intelligence; WCST, Wisconsin Card Sorting Test; WISC, Wechsler Intelligence Scale for Children; WMS-III, Wechsler Memory Scale; WRAT, Wide Range Achievement Test

### Characteristics of the database

Data were extracted from 3 studies (detailed in Table 1) for a total sample size of 151 CHR-P patients [mean (SD) age, 16.48 (2.41) years; 66 (32.45%) were female] and 64 HC individuals [mean (SD) age, 16.79 (2.38) years; 27 (42.18%) were female].

### Neurocognitive functioning in individuals at CHR-P compared with HC individuals

In Bowie et al. [37] study a worse functioning was seen at baseline in all the CHR-P subgroups—one subgroup receiving treatment with antidepressants, other with second-generation antipsychotics and the last group remained medication-naïve—in comparison with healthy controls (HC). Nevertheless, since different treatments were studied at follow-up some differences could be appreciated, as post hoc comparisons showed an improvement in California Verbal Learning Test Total Learning (CVLT Total Learning) for the group taking antidepressants (AD) compared to the group in treatment with antipsychotics (AP) (*F* (1, 26) = 5.6, *p* = 0.027). The medication-naïve CHR-P group had small effect size changes in CVLT Total Learning (*d* = 0.39), whereas the AD group had moderate to large effect size improvements in the CVLT Total Learning (*d* = 0.63). Conversely, the AP group had small size effects indicating a worsening performance on the CVLT Total Learning (*d* =  − 0.33) [36]. Similar results were found by Carrion et al. [38] and Woodberry et al. [39], finding stable scores in verbal memory in the HC group relative to CHR-P individuals, who had lower scores at baseline—although it was not statistically significant—and worsened at follow-up.

Sustained attention was analyzed in the three studies [37–39] through Continuous Performance Test—Identical Pairs (CPT-IP). Two of them [38, 39] did not find significant differences between HC and CHR-P groups. Carrion et al. stated that all subject groups in their study demonstrated similar and small improvements—probably due to practice effects—in performance in many of the domains, but they found significant improvements in sustained attention.

Bowie et al. [37] found an improvement in the AD group relative to the AP group (*F* (1, 21) = 11.0, *p* = 0.003), and also medication free group compared to the AP group (*F* (1, 29) = 10.4, *p* = 0.003). The group that remained off medication and the AD group had both moderate to large effect size changes on the Cognitive Performance Test digits (CPT digits) (*d* = 0.80 and *d* = 1.02, respectively). The AP group had small effects indicating worsening performance on CPT digits (*d* =  − 0.39).

When studying executive functioning, the Wisconsin Card Sorting Test (WCST) showed that CHR-P groups performed significantly below relative to HCs.

Regarding to verbal fluency, only Bowie et al. [37] gave raw results, in which stable scores were obtained in Controlled Oral Word Association Test (COWAT) between baseline and follow-up assessment, with the exception of the group taking second-generation AP, which revealed a non-statistically significant declining. The rest of the studies did not show separate verbal fluency results.

When it comes to processing speed Carrión et al. [38] showed an impairment at baseline in CHR-P patients that persisted after psychosis onset and did not further deteriorate, while HC performed stably. Bowie et al. [37] suggested a possible relationship between results in processing speed—Trails Making Test Part A—and executive functions—Trails Making Test Part B—and negative and disorganized symptoms.

Even though included studies studied more neurocognitive domains and tasks, due to the lack of raw scores, there is not sufficient data to make an overall description of other domains, such as working memory (LNS), and premorbid IQ (WRAT-3).

### Neurocognitive functioning in individuals at CHR-P associated with transition to psychosis

Two studies registered transition to psychosis and its relationship with neurocognitive impairment. One of them [39] registered a transition rate of 18.86% with a mean (SD) time to transition of 6.3 (6.8) months, whereas the second [38] found a transition rate of 33.33% with a mean (SD) time to transition to psychosis of 12.34 (16.06) months. Woodberry et al. [39] did not find a significantly bigger overall neurocognitive impairment at follow-up for CHR-P who transitioned to psychosis relative to those who did not. They conducted an exploratory comparison of effect sizes of the standardized residuals of executive functions and memory domains and did find differences in memory, finding larger effects sizes in individuals who transitioned to psychosis (Cohen's *d* =  − 1.89, CI − 2.71 to − 1.08) in contrast with those who did not (Cohen's *d* =  − 0.61, CI − 1.08 to − 0.14).

In contrast, Carrion et al.[38] found that CHR-P individuals that transitioned to psychosis presented a substantial impairment in global neurocognitive and intellectual performance from baseline to follow-up assessment in contrast with those who did not transition and with the HC group. Nevertheless, similar to the sample of Woodberry et al. [39], no differences were found in executive functioning. In the same way, results concerning language domain did not show relevant differences between these groups. CHR-P individuals that transitioned revealed small effect sizes of improvement in neurocognitive performance compared to those who did not, with the exception of processing speed and attention domains, in which medication-free not transitioned CHR-P subjects had moderate to large improvements, while those who transitioned to psychosis showed smaller improvements.

In addition, in the work of Carrion et al. [38] neurocognitive performance of the CHR-P group could be assessed on average 8 months after the onset of fully psychosis. In this way, it was found out that this global impairment presented in CHR-P subjects who developed psychosis persisted stable over the course of the follow-up, with no appreciable deterioration after the onset of psychosis. On the other hand, during this follow-up period CHR-P subjects that did not develop psychosis revealed mild to no impairments in neurocognitive and intellectual performance independent of medication treatment.

### Relationship between neurocognitive functioning and clinical symptomatology

Bowie et al. studied possible correlations between neurocognitive and psychopathology scores at baseline, and they found small associations and inconsistencies in their direction, not finding statistical significance after Bonferroni correction. In the same way, there was no significant correlation between neurocognitive changes and small changes following treatment in the attenuated positive, attenuated negative, disorganized, depressive, and anxiety symptoms during follow-up [37].

In contrast, in the sample studied by Carrion et al., an increase in the severity of positive symptoms was related to improvements in processing speed, sustained attention, working memory, and global cognition. Similarly, improvement in working memory and global cognition were related to more negative symptoms and disorganized symptoms, respectively. Nevertheless, these relationships were not consistent after multiple comparisons [38].

## Discussion

One of the main findings of this review is the common finding of worse outcomes in CHR-P individuals in verbal learning, sustained attention and executive functioning domains when compared with HC individuals. Impairments in these domains have previously been studied, and our findings match with already performed meta-analysis in the adult CHR-P population [22, 27, 40, 41], which showed a widespread impairment of neurocognitive functioning, albeit to changing severity depending on the domain. In the same way, impairments in these domains have been described as some of the core cognitive deficits in schizophrenia [42, 43]. Particularly, sustained visual attention—measured by the CPT-IP—has been found to be heritable, reliable, and stable across the evolution of the disorder [44], opening a new pathway of research in schizophrenia.

When it comes to verbal learning, Becker et al. [45] found stable deficits as assessed by the CVLT-Trials 1–5, free recall total correct—in a CHR-P population before and after the onset of psychosis in contrast with HCs and a meta-analysis comparing neurocognitive functioning in first-episode psychosis [46] showed that performance on verbal memory—CVLT—was among the poorest compared to HCs. Consequently, the results found in attention and verbal learning are key findings, as those domains have also been considered reliable predictors of conversion from clinical high-risk state to psychosis [47–50].

Processing speed plays a central role in a wide range of high-order cognitive abilities, such as language learning and visual scanning, in the earliest phases of a psychotic disorder [51]. Besides, because of the variety of task demands used in processing speed measures, deficits in this domain are mostly likely to show a widespread and diffuse neurocognitive dysfunction in different but interconnected brain regions and may moderate results of other neurocognitive domains that are usually associated with pathophysiology [52].

In Woodberry et al. [39], no neurocognitive impairment was demonstrated when comparing CHR-P that transitioned to psychosis with those who did not. Nevertheless, it is a fact that the 1-year effect size for verbal memory test raw scores for the group that later transitioned was large (*d* =  − 1.24) and reminded of results found in first-episode psychosis populations [46]. Moreover, this transitioned group revealed baseline impairments in estimated IQ, reminding also first-episode psychosis samples [46]. There was a lack of progressive impairment at follow-up. Therefore, these results could suggest that IQ impairment identified in schizophrenia samples may already be before the onset of full-blown psychosis.

In line with this, the results of the report of Carrion et al. [38] revealed that cognition is impaired prior to the onset of psychosis and that the onset of psychosis does not have a deleterious effect on the course of neurocognition. Thus, it seems that neurocognitive impairments represent trait risk markers which might work as predictors of psychosis prior to the onset, and suggests that cognitive impairment during the CHR-P state is related to the vulnerability to disorder, which is consistent with the neurodevelopmental model [53].

In the two reports that studied correlations between clinical and neurocognitive changes [37, 38], weak associations were found, consistent with previous reports in adults with schizophrenia [54]. Thus, psychopathological symptoms and neurocognition are distinct features in populations at high clinical risk for psychosis.

Bowie et al. [37] found that the use of AD medication over a 6 months was associated with improvements in verbal learning and sustained attention and that treatment with second-generation AP had a deleterious effect on those cognitive domains. These findings are consistent with a previous work that stated that treatment adherence was better and transition to psychosis was less likely when CHR-P adolescents were treated with AD compared to second-generation neuroleptics [55]. This finding goes along with reports related to the use of antidepressant in other clinical populations [56, 57]. However, there is no clear evidence that medications can impact the course of psychosis onset in CHR-P samples [58, 59]. Nevertheless, a meta-analytic review on this topic only revealed significant positive effects on psychomotor speed and delayed recall [60].

When comparing neurocognitive impairments between adolescents and adults, it has been revealed that adolescents at CHR-P showed more significant impairments and were associated with a higher risk of conversion to psychosis [61]. In fact, the adolescent CHR-P group showed a wider range of neurocognitive dysfunction. Moreover, in the same report, it was found that only Brief Visuospatial Memory Test-Revised (BVMT-R) test showed significant differences between the CHR-P adolescent and adult group, demonstrating a better performance in adolescents. This study stated that neurocognitive assessments for predicting conversion were more accurate in adolescents than adults, suggesting that neurocognitive developmental pathways may play a more relevant role in adolescent-onset psychosis.

When focusing only in adult populations current evidence reveals, first, a general dysfunction of neurocognitive functioning in individuals at CHR-P [22, 62, 63] and second, the fact that baseline neurocognitive impairments in verbal learning, visual memory, processing speed, attention and general intelligence are associated with longitudinal risk of developing psychosis [22].

Nevertheless, these results are still not generalizable to the children and adolescent population. On one hand, due to the investigation data gap in this area, and on the other hand, different variables might hamper these results coming to light, such as the increased brain plasticity in adolescents [64].

For these reasons, it is worthwhile to reference studies that meet the established inclusion criteria and have a sample mean age close to the established age range. Although the findings from these studies cannot be directly generalized to the target population, they provide valuable insights into potential outcomes for younger patients.

Several studies have shown that young individuals at CHR-P present impaired executive functions, attention, working memory, processing speed, verbal fluency, verbal memory, and visual memory at baseline, compared with healthy controls [65–68]. Moreover, individuals that later develop psychosis show significantly bigger impairment in contrast with non-converters in those areas [67, 68]. At follow-up, slowed processing speed has been described in CHR-P samples [65, 66]. When comparing the CHR-P with other groups with a lower risk of transition to psychosis (intermediate and marginal-risk groups), processing speed impairment was common across all groups [66]. Earlier studies have proposed that functional capacity in schizophrenia is primarily influenced by processing speed and is a mediator between verbal memory, verbal fluency, and overall functioning [69]. Thus, it could be cautiously interpreted that impairment in processing speed is an early alteration in pre-psychotic states. It could serve as a risk marker in very early stages that could trigger impairment in other neurocognitive areas.

A number of neurocognitive domains, such as attention, processing speed, visual memory, verbal memory and verbal fluency, exhibit different longitudinal changes in individuals who develop psychosis compared to those who do not. The former does not show changes from baseline to follow-up, while the latter typically shows improvements in these domains [67, 68, 70, 71]. Moreover, digit-symbol coding has been described to worsen in individuals that transited and to improve in those that remain in CHR-P state at 1-year follow-up [70]. In some samples, neurocognitive performance in CHR-P that converted to psychosis did not reveal any statistically significant difference compared to FEP. Among those who did not develop psychosis, the subgroup that later remitted from the initial CHR-P state showed great similarity with healthy controls at follow-up [72].

Particularly, verbal fluency has been studied in association with clinical symptomatology and functionality. An improvement in verbal fluency at follow-up has been associated with a significant reduction in positive symptoms [71]. The magnitude of change in this domain has been associated with changes in negative symptoms and functioning in CHR-P individuals [73]. Furthermore, over a 2-year follow-up, CHR-P individuals who achieved remission showed a significant improvement in their performance on verbal fluency tasks, while those who did not achieve remission experienced a decline in their performance on these tests [72]. In parallel with other domains, individuals that transitioned to psychosis had stable verbal fluency outcomes over time, while those who did not transition CHR-P showed some improvement [67]. Regarding literature, earlier studies have indicated that a decline in semantic fluency may be an early risk indicator for schizophrenia [74]. Furthermore, verbal fluency has shown a stronger correlation with community functioning than other neurocognitive domains [75].

## Limitations

There are several limitations to this review. First, the lack of studies meeting the inclusion criteria makes the generalizability of the findings difficult. The age limit (sample mean age ≤ 18 years) was the main reason for exclusion during the screening. Studying children, adolescents and adults altogether reflects that existing evidence studying neurocognition in CHR-P populations treats them in the same manner regardless their developmental state, suggesting that cognitive dysfunctions in all age groups have the same meaning in prognosis, risk factor identification or prediction of transition to psychosis. Thus, this viewpoint ignores age-related effects on the psychosis onset, as well as neurodevelopmental changes occurring in adolescence—myelination and synaptic pruning. Those processes are known to be altered when early onset psychosis comes out; for instance, by alterations of neurons in anterior cingulate cortex and prefrontal cortex that increase their myelination during adolescence [21, 65, 66]. As it seems that a worsening or improvement of neurocognitive functions at varying stages of the disorder lead to different outcomes, further study should be performed to improve the efficiency of early identification and accuracy of prediction in early onset psychosis.

Considering the prognostic limitations of the CHR-P design is also relevant, since some CHR-P individuals might not transition to a psychosis state. Therefore, using accurate instruments to sharpen and adjust to the real clinical situation is relevant. In fact, current evidence has demonstrated that CHR-P assessment instruments have good prognostic accuracy predicting psychosis, comparable to the accuracy of preventive approaches in other areas of medicine [67].

Another limitation is that some of the included studies excluded individuals with BLIPS. On one hand, the clinical heterogeneity in this CHR-P subgroup over time makes it difficult to evaluate the course of the disorder properly without prospective assessments. Besides, some authors do not consider BLIPS as CHR-P subjects, since, by definition, these individuals have already experienced psychotic symptoms and, therefore, are not a risk-state any more. This way, the generalizability of the results found in these reports should be taken cautiously.

Individuals meeting criteria for GRFD also raise a relevant question that may affect the generalizability of the results, since there are offspring of parents with psychosis that do not completely meet criteria for GRFD. Nevertheless, they are also at lifetime risk of developing major psychopathology, even though it is not an imminent risk [79]. It is already known that offspring of parents with psychosis are at a higher risk of developing the disorder themselves [80, 81]. Moreover, there is evidence that individuals at genetic risk for psychosis have difficulties in verbal memory, attention and executive functions [9, 63, 81]. Certain similarities between these two populations could be predicted. However, it's cautious to wait to acquire more evidence on neurocognition in both types of individuals and to explore more deeply the differences in psychopathology and prognosis between them.

Moreover, studies with longer follow-up periods should be considered, as a CHR-P state might last longer than the follow-up and be improperly considered not transitioned. Furthermore, longer follow-up periods would help on minimizing practice effects and, thus, obtain more accurate results.

## Conclusions

In summary, this review found clearly worse performance in CHR-P individuals compared to HC in verbal learning, sustained attention and executive functioning in adolescent population. In children and adolescents, neurocognition may be already impaired before the psychosis onset and remains stable during the transition to psychosis. Further study should be performed to obtain more robust evidence in this population.

## Supplementary Information

Below is the link to the electronic supplementary material.

Supplementary file 1 (DOCX 58 kb)

## Data Availability

The data that support the findings of this study are available from the corresponding author upon request.
