# Correction: Longitudinal change in neurocognitive functioning in children and adolescents at clinical high risk for psychosis: a systematic review

**DOI:** 10.1007/s00787-024-02621-5

**Published:** 2024-12-21

**Authors:** Borja Pedruzo, Claudia Aymerich, Malein Pacho, Jon Herrero, María Laborda, Marta Bordenave, Anthony J. Giuliano, Robert A. McCutcheon, Luis Gutiérrez-Rojas, Philip McGuire, William S. Stone, Paolo Fusar-Poli, Miguel Ángel González-Torres, Ana Catalan

**Affiliations:** 1 Department of Psychiatry, Basurto University Hospital, Bilbao, Spain; 2 Worcester Recovery Center and Hospital, Massachusetts Department of Mental Health, Boston, USA; 3 Department of Psychosis Studies, Institute of Psychiatry, Psychology and Neuroscience, King’s College London, London, UK; 4 Department of Psychiatry, University of Oxford, Oxford, UK; 5 Psychiatry Service, San Cecilio University Hospital, Granada, Spain; 6 National Institute for Health Research Biomedical Research Centre, London, UK; 7 Outreach and Support in South London Service, South London and Maudsley National Health Service Foundation Trust, London, UK; 8 Department of Psychiatry, Beth Israel Deaconess Medical Center, Harvard Medical School, Boston, MA USA; 9 Early Psychosis: Interventions and Clinical-Detection (EPIC) Lab, Department of Psychosis Studies, Institute of Psychiatry, Psychology and Neuroscience, King’s College London, London, UK; 10 Department of Brain and Behavioral Sciences, University of Pavia, Pavia, Italy; 11 Neuroscience Department, University of Basque Country (UPV/EHU), Leioa, Spain; 12 Biocruces Bizkaia Health Research Institute, Barakaldo, Spain; 13 CIBERSAM. Centro Investigación Biomédica en Red de Salud Mental, Madrid, Spain

**Correction: European Child & Adolescent Psychiatry (2023) 33:3377–3387** 10.1007/s00787-023-02221-9

In this article the affiliation details for Author Borja Pedruzo were incorrectly given as “Department of Psychiatry, Basurto University Hospital, Bilbao, Spain” but should have been “Department of Psychiatry, Basurto University Hospital, Bilbao, Spain and Neuroscience Department, University of Basque Country (UPV/EHU), Leioa, Spain”.

The original article has been corrected.

