# Supplementary material for: Longitudinal change in neurocognitive functioning in children and adolescents at clinical high risk for psychosis: a systematic review
Source: Eur Child Adolesc Psychiatry. 2023 May 18;33(10):3377–87. doi: 10.1007/s00787-023-02221-9 (PMC11564316; doi:10.1007/s00787-023-02221-9)
Supplement: Supplementary file 1 — (DOCX 58 kb) [file 787_2023_2221_MOESM1_ESM.docx]

SUPPLEMENTARY MATERIAL

**Methods S1**. Search terms used in the literature search

The search terms were: children” OR “adolescent” OR “adolescence” AND “longitudinal” OR “follow-up” AND "cognit*" OR "neurocognit*" OR "social cognit*" OR “neuropsy*” AND "psychosis risk" OR "prodrom*" OR "ultra-high risk" OR "clinical high risk" OR "genetic high risk" OR "at risk mental state" OR "at-risk mental state" OR "basic symptoms".

**Methods S2**. Types of CHR-P psychometric interviews included (modified from Fusar- Poli et al. 2020[1])

The CHR-P state comprises the Clinical High Risk for Psychosis (CHR-P) state and/or the Basic Symptoms (BS) [1].

The following CHR-P psychometric interviews were considered to define the UHR state: Comprehensive Assessment of At-Risk Mental States (CAARMS)[2], Structured Interview for Psychosis-risk Syndromes (SIPS) [3,4], Basel Screening Instrument for Psychosis (BSIP) [5] and Early Recognition Inventory (ERIraos) [6].

The following CHR-P psychometric interviews were considered to define the BS1: Bonn Scale for the Assessment of Basic Symptoms (BSABS) [7], Schizophrenia Proneness Instrument -Adult (SPI-A) and Child and Youth (SPI-CY) version [8].

Furthermore, we considered early operationalisations of the CHR-P state, which were based on the Positive and Negative Syndrome Scale (PANSS) [9] and Brief Psychiatric Rating Scale (BPRS) [10].

**Methods S3.** Neurocognitive domains considered in the current meta-analysis (7 MATRICS domains and 8 CHR-P domains. Adapted from Fusar-Poli et al. 2012 [11] and Hauser et al. 2017 [12]. CHR-P, Clinical High Risk for Psychosis.

As a note, the MATRICS was not designed to be a comprehensive battery for the assessment of cognition but rather was developed with a narrower purpose of creating a brief, highly portable and tolerable consensus battery of neurocognitive domains most likely to be sensitive to both the most common (or best characterized) cognitive impairments in schizophrenia spectrum disorders

and to change following targeted treatment (pharmacological or psychological). In fact, the range of cognitive impairment in schizophrenia spectrum disorders is broader and more complex than indexed by the MATRICS, and we should expect no less of its putative clinical high-risk states. As such, in an effort to produce a comprehensive review of the extant literature, we endeavoured to be more inclusive in our approach and thus included additional neurocognitive domains and tasks that have been employed in cognitive studies of CHR-P to date and which are frequently assessed in clinical settings. While the MATRICS represents an organizing framework, it is necessarily limited such that any comprehensive meta-analysis requires the inclusion of a broader set of neurocognitive domains and tests that adhere to a related but non-redundant categorizing scheme.

| **Neurocognitive**  **domains** | **Tasks** |
| --- | --- |
| ***MATRICS domains*** | |
| **Processing Speed** | - Trail Making Test-Part A (TMT-A) [13] - Brief Assessment of Cognition Scale Symbol Coding (BACS SC) [14] - Animal Fluency[15] - Letter Fluency [16] - Digit symbol coding test (DST) [17] - Stroop color word reading (Stroop W) [18] - Stroop color naming task (Stroop C) [18] |
| **Attention/Vigilance** | - Continuous Performance Test – Identical Pairs (CPT-IP)* [19,20] |
| **Working Memory** | - Wechsler Memory Scale-III Spatial Span Subtest (WMS-III: SS) [17] - Letter Number Span (LNS) [21] - Letter Number Sequencing Test (LNST)[17] - Arithmetic (any WAIS) [17] - Self-Ordered Pointing Task (SOPT)[22] |
| **Verbal Learning** | - Hopkins Verbal Learning Test—Revised (HVLT-R)** [23] - Rey Auditory Verbal Learning Test (RAVLT)*** [24] - California Verbal Learning Test I/II (CVLT)**** [24–26] - Wide Range Achievement Test – III (WRAT-III) Reading **reference en Woodberry** [27] |
| **Visual learning** | - Brief Visuospatial Memory Test-Revised (BVMT-R)** [28] - Wechsler Memory Scale Immediate Visual Memory (WMS VM) [17] - Rey–Osterrieth Complex Figure test Immediate Recall (ROCF) [29] |
| **Reasoning and**  **Problem-Solving** | - Neuropsychological Assessment Battery Mazes (NAB Mazes) [30] |
| **Social cognition+** | - Reading the Mind in the Eyes Test (RMET)[31,32] - Degraded Facial Affect Recognition (DFAR) [33] - Hinting [34] |
| ***CHR-P domains*** | |
| **General intelligence IQ** | - Wechsler Adult Intelligence Scale- 3^rd^ edition (WAIS-III) [17] - Wechsler Adult Intelligence Scale-Revised (WAIS-R) [35] - Wechsler Intelligence Scale for Children- 3^rd^ edition (WISC-III) [36] |
| **Premorbid IQ** | - National Adult Reading Test (NART)[37] - MehrfachWortschaftz-Intelligenz Test-part B (MWT-B)[38] |

| **Visuospatial ability** | - WAIS/WISC Block Design (WAIS/WISC BD) [17,36] |
| --- | --- |
| **Verbal fluency** | - Controlled Oral Word Association Test (COWAT) [39] |
| **Verbal memory** | - RAVLT Delayed Recall (RAVLT DR) [24] |
| **Visual memory** | - ROCF Delayed Recall (ROCF DR) [29] - Wechsler Memory Scale Visual Reproduction Delayed Recall (WMS VR) [17] |
| **Executive functioning** | - Trail Making Test- Part B (TMT-B) [13] - Wisconsin Card Sorting Test (WCST): categories, number of correct responses, perseverative errors and perseverative responses[40] - Stroop Test: Interference [41] |
| **Motor functioning** | - Finger Tapping Test (Tapping) [42] |
| **Olfaction** | - University of Pennsylvania Smell Identification Test (UPSIT) [43] |

**^+^**Social cognition encompassed: (a) emotional processing, (b) social perception and knowledge, (c) theory of mind, and (d) attributional

bias). *****Mean d' across conditions; **Total Learning Trials 1‐3; ***Learning Trials; **** Trials 1-5 Total Correct Only tasks with 3 or more available studies in the dataset are listed

**Methods S4.** Extracted variables

Author, year, follow-up months, type of CHR-P psychometric instrument, number of CHR-P baseline, number of healthy controls at baseline, age at baseline (median and SD), range age, male sex %, years of education, white race %, antipsychotic treatment exposure at baseline, cognition domain, the task used, results of the neurocognitive task (mean and SD), number of CHR-P who transitioned to psychosis, time to transition, follow-up time (months), positive psychotic and negative symptoms at baseline and at follow-up, functioning status at baseline and at follow-up, andNOS quality.

**Methods S5**. Glossary of terms

- AD: Antidepressant
- AP: Antipsychotic
- BACS SC: Brief Assessment of Cognition Scale Symbol Coding
- BVMT-R: Brief Visuospatial Memory Test-Revised
- CHR-P: Clinical High Risk for Psychosis
- CPT-IP: Continuous Performance Test – Identical Pairs
- CVLT: California Verbal Learning Test I/II
- DST: Digit symbol coding test
- FEP: First Episode Psychosis
- FU: Follow-up
- HC: Healthy Controls
- HVLT-R: Hopkins Verbal Learning Test—Revised
- LNS: Letter Number Span
- Mo: Months
- NAB Mazes: Neuropsychological Assessment Battery Mazes
- NOS: Newcastle-Ottawa Scale
- SD: Standard Deviation
- Tapping: Finger Tapping Test
- TMT-A and TMT-B: Trail Making Test Part A and B
- WAIS-III: Wechsler Adult Intelligence Scale- 3rd edition
- WAIS-R: Wechsler Adult Intelligence Scale-Revised
- WCST: Wisconsin Card Sorting Test
- WISC-III: Wechsler Intelligence Scale for Children- 3rd edition
- WMS-III: SS: Wechsler Memory Scale-III Spatial Span Subtest

**Table S1**. PRISMA statement and checklist

| **Section/topic** | **#** | **Checklist item** | **Page** |
| --- | --- | --- | --- |
| **TITLE** | | |  |
| Title | 1 | Identify the report as a systematic review, meta-analysis, or both | *Cover page* |
| **ABSTRACT** | | |  |
| Structured summary | 2 | Provide a structured summary including, as applicable: background; objectives; data sources; study eligibility criteria, participants, and interventions; study appraisal and synthesis methods; results; limitations; conclusions and implications of key findings; systematic review registration number | *Abstract* |
| **INTRODUCTION** | | |  |
| Rationale | 3 | Describe the rationale for the review in the context of what is already known | *Introduction* |
| Objectives | 4 | Provide an explicit statement of questions being addressed with reference to participants, interventions, comparisons, outcomes, and study design (PICOS) | *Introduction* |
| **METHODS** | | |  |
| Protocol and registration | 5 | Indicate if a review protocol exists, if and where it can be accessed (e.g. Web address), and, if available, provide registration information including registration number | *Methods* |
| Eligibility criteria | 6 | Specify study characteristics (e.g. PICOS length of follow-up) and report characteristics (e.g. years considered, language, publication status) used as criteria for eligibility, giving rationale | *Methods* |
| Information sources | 7 | Describe all information sources (e.g., databases with dates of coverage, contact with study authors to identify additional studies) in the search and date last searched | *Methods* |
| Search | 8 | Present full electronic search strategy for at least one database, including any limits used, such that it could be repeated | *Methods* |
| Study selection | 9 | State the process for selecting studies (i.e. screening, eligibility, included in systematic review, and, if applicable, included in the meta-analysis) | *Methods* |
| Data collection process | 10 | Describe method of data extraction from reports (e.g. piloted forms, independently, in duplicate) and any processes for obtaining and confirming data from investigators | *Methods* |
| Data items | 11 | List and define all variables for which data were sought (e.g. PICOS funding sources) and any assumptions and simplifications made | *Methods* |

| Risk of bias in individual studies | 12 | Describe methods used for assessing risk of bias of individual studies (including specification of whether this was done at the study or outcome level), and how this information is to be used in any data synthesis | *Methods* |
| --- | --- | --- | --- |
| Summary measures | 13 | State the principal summary measures | *Methods* |
| Risk of bias across | 15 | Specify any assessment of risk of bias (i.e. Newcastle-Ottawa Scale (NOS)), that may affect the cumulative evidence | *Methods* |
| Additional analyses | 16 | Describe methods of additional analyses (e.g. sensitivity or subgroup analyses, meta-regression), if done, indicating which were pre-specified | *Methods* |
| **RESULTS** |  |  |  |
| Study selection | 17 | Give numbers of studies screened, assessed for eligibility, and included in the review with reasons for exclusions at each stage, ideally with a flow diagram | *Results* |
| Study characteristics | 18 | For each study, present characteristics for which data were extracted (e.g. study size, PICOS follow-up period) and provide the citations | *Results* |
| Risk of bias within studies | 19 | Present data on risk of bias of each study and, if available, any outcome level assessment (see item 12) | *Results* |
| Results of individual studies | 20 | For all outcomes considered (benefits or harms), present for each study a summary data for each intervention group | *Results* |
| Synthesis of results | 21 | Present results of analyses | *Results* |
| Risk of bias across studies | 22 | Present results of any assessment of the risk of bias across studies (see Item 15) | *Results* |
| Additional analysis | 23 | Give results of additional analyses, if done (e.g. sensitivity or subgroup analyses, meta-regression see Item 16) | *Results* |
| **DISCUSSION** |  |  |  |
| Summary of evidence | 24 | Summarize the main findings including the strength of evidence for each main outcome; consider their relevance to  key groups (e.g. healthcare providers, users, and policymakers) | *Discussion* |
| Limitations | 25 | Discuss limitations at study and outcome level (e.g. risk of bias), and at review-level (e.g. incomplete retrieval of identified research, reporting bias) | *Discussion* |
| Conclusions | 26 | Provide a general interpretation of the results in the context of other evidence, and implications for future research | *Discussion* |
| **FUNDING** |  |  |  |
| Funding | 27 | Describe sources of funding for the systematic review and other support (e.g. supply of data), role of funders for the systematic review | *Funding* |

**Table S2**. MOOSE checklist

| **Criteria** | | **Brief description of how the criteria were handled in the meta-analysis** |
| --- | --- | --- |
| **Reporting of background should include** | |  |
| √ | Problem definition | To examine at a meta-analytical level whether neurocognitive deficits are evident in Clinical High Risk (CHR-P) for psychosis children and adolescents to preferably healthy controls (HC) and to define the specific pattern of these neurocognitive deficits. To identify neurocognitive impairments that specifically predicted the later transition to psychosis in the CHR-P population |
| √ | Hypothesis statement | We hypothesized that CHR-P state would have a significant impairment in neurocognitive domains, especially those who develop psychosis. |
| √ | Description of study outcomes | In line with our earlier meta-analysis the different neurocognitive tasks were grouped in neurocognitive domains on the basis of the criteria developed by the MATRICS conference and then discussed by us, according to the indications of the articles included: (1) processing speed, (2) verbal learning, (3) working memory, (4) reasoning and problem-solving, (5) visual learning, (5) attention and vigilance, and (7) social cognition. Further, we have analysed the CHR-P domains of (8) general intelligence, (9) premorbid intelligence, (10) visuospatial ability, (11) verbal memory, (12) visual memory, (13) executive functioning, (14) motor functioning, and (15) olfaction. We reported differences between CHR-P population and HC in these domains, measured by standardised scales. |
| √ | Type of exposure or intervention used | We included individual studies that reported neurocognitive data in CHR-P population. |
| √ | Type of study designs used | Case-control studies, and cohort studies, which investigate the longitudinal neurocognitive functioning CHR-P for psychosis compared toHC. |
| √ | Study population | CHR-P state. |
| **Reporting of search strategy should include** | |  |
| √ | Qualifications of researchers | The credentials of the investigators are indicated in the author list and in the acknowledgements. |
| √ | Search strategy. including time period included in the synthesis and keywords | We performed a multi-step literature search using the following keywords: children” OR “adolescent” OR “adolescence” AND “longitudinal” OR “follow-up” AND "cognit*" OR "neurocognit*" OR "social cognit*" OR “neuropsy*” AND "psychosis risk" OR "prodrom*" OR "ultra-high risk" OR "clinical high risk" OR "genetic high risk" OR "at risk mental state" OR "at-risk mental  state" OR "basic symptoms" from inception until 15th July 2022. |
| √ | Databases and registries searched | Web of Science database (Clarivate Analytics): Web of Science Core Collection, BIOSIS Citation Index, KCI-Korean Journal Database, MEDLINE, Russian Science Citation Index, PubMed and SciELO Citation Index. |
| √ | Use of hand searching | We hand-searched bibliographies of retrieved papers for additional references. |
| √ | List of citations located and those excluded. including justifications | Details of the literature search process are outlined in the results section and in the PRISMA flow-chart. |

| √ | Method of addressing articles published in languages other than English | Only articles in English language were selected. |
| --- | --- | --- |
| √ | Method of handling abstracts and unpublished studies | Original individual studies were included. Conference proceedings, reviews, editorials, clinical cases and unpublished studies were excluded. |
| √ | Description of any contact with authors | A description of the contact with corresponding authors to request additional data for this study is detailed in methods  section. |
| **Reporting of methods should include** | |  |
| √ | Description of relevance or  appropriateness of studies assembled for assessing the hypothesis to be tested | Detailed inclusion and exclusion criteria were described in the methods section. |
| √ | Rationale for the selection and coding of data | Data extracted from each of the studies were relevant to the population characteristics, study design, comparison group, exposure and outcomes. |
| √ | Assessment of confounding factors | Confounding factors were systematically assessed in each neurocognitive domain. |
| √ | Assessment of study quality | We adapted the Newcastle-Ottawa Scale for the evaluation of cross-sectional and cohort studies. |
| √ | Assessment of heterogeneity | Heterogeneity was assessed with the I^2^ index. |
| √ | Description of statistical methods in  sufficient detail to be replicated | Statistical methods are described in detail in the methods section. |
| √ | Provision of appropriate tables and graphics | We included the PRISMA flow-chart and several tables and graphics to describe the literature search and our results. |
| **Reporting of results should include** | |  |
| √ | Graph summarizing individual study estimates and overall estimate | We have appended them in the main text. Additional graphs were presented as supplementary material to fully describe the results. |
| √ | Table giving descriptive information for each study included | We have presented descriptive information for each study in the supplementary material. |
| √ | Results of sensitivity testing | Subgroup analyses were conducted to analyse differences between used task in each neurocognitive domain. |
| √ | Indication of statistical uncertainty of findings | We reported mean estimates for the main outcome and 95% CI. |
| **Reporting of discussion should include** | |  |
| √ | Quantitative assessment of bias | Publication biases were assessed by funnel plots visual inspections and Egger test ^49^. The trim and fill methods were used as sensitivity analyses to correct biases if detected. |
| √ | Justification for exclusion | Exclusion criteria and justification are described in the manuscript. |
| √ | Assessment of quality of included studies | We adapted the Newcastle-Ottawa Scale for the evaluation of cross-sectional and cohort studies. |
| **Reporting of conclusions should include** | |  |
| √ | Consideration of alternative explanations for observed results | We discussed other explanations for our findings in the discussion section. |

| √ | Generalization of the conclusions | We have addressed the generalization of the conclusions in the discussion section. |
| --- | --- | --- |
| √ | Guidelines for future research | We have suggested possible streams of future development and research in the discussion. |
| √ | Disclosure of funding source | Funding source described at the end of the manuscript. No separate funding was necessary for the undertaking of this meta- analysis. |

**References**

1. Fusar-Poli P, Salazar de Pablo G, Correll CU, Meyer-Lindenberg A, Millan MJ, Borgwardt S, et al. Prevention of Psychosis: Advances in Detection, Prognosis, and Intervention. JAMA Psychiatry. 1 de julio de 2020;77(7):755-65.

2. Yung AR, Yuen HP, McGorry PD, Phillips LJ, Kelly D, Dell’Olio M, et al. Mapping the onset of psychosis: the Comprehensive Assessment of At-Risk Mental States. Aust N Z J Psychiatry. diciembre de 2005;39(11-12):964-71.

3. Fusar-Poli P, Cappucciati M, Rutigliano G, Lee TY, Beverly Q, Bonoldi I, et al. Towards a Standard Psychometric Diagnostic Interview for Subjects at Ultra High Risk of Psychosis: CAARMS versus SIPS. Psychiatry J. 2016;2016:7146341.

4. The Psychosis-Risk Syndrome: Handbook for Diagnosis and Follow-Up.

5. Riecher-Rössler A, Aston J, Ventura J, Merlo M, Borgwardt S, Gschwandtner U, et al. [The Basel Screening Instrument for Psychosis (BSIP): development, structure, reliability and validity]. Fortschr Neurol Psychiatr. abril de 2008;76(4):207-16.

6. Häfner H, Maurer K, Ruhrmann S, Bechdolf A, Klosterkötter J, Wagner M, et al. Early detection and secondary prevention of psychosis: facts and visions. Eur Arch Psychiatry Clin Neurosci. abril de 2004;254(2):117-28.

7. Vollmer-Larsen A, Handest P, Parnas J. Reliability of measuring anomalous experience: the Bonn Scale for the Assessment of Basic Symptoms. Psychopathology. 2007;40(5):345-8.

8. Fux L, Walger P, Schimmelmann BG, Schultze-Lutter F. The Schizophrenia Proneness Instrument, Child and Youth version (SPI-CY): practicability and discriminative validity. Schizophr Res. mayo de 2013;146(1-3):69-78.

9. Kay SR, Fiszbein A, Opler LA. The positive and negative syndrome scale (PANSS) for schizophrenia. Schizophr Bull. 1987;13(2):261-76.

10. Overall, J.E. and Gorham, D.R. The brief psychiatric rating scale (BPRS): Recent developments in ascertainment and scaling. Psychopharmacology Bulletin. 1988; 24, 97-99.

11. Fusar-Poli P, Deste G, Smieskova R, Barlati S, Yung AR, Howes O, et al. Cognitive Functioning in Prodromal Psychosis: A Meta-analysis. Arch Gen Psychiatry [Internet]. 1 de junio de 2012 [citado 11 de septiembre de 2022];69(6). Disponible en: http://archpsyc.jamanetwork.com/article.aspx?doi=10.1001/archgenpsychiatry.2011.1592

12. Hauser M, Zhang JP, Sheridan EM, Burdick KE, Mogil R, Kane JM, et al. Neuropsychological Test Performance to Enhance Identification of Subjects at Clinical High Risk for Psychosis and to Be Most Promising for Predictive Algorithms for Conversion to Psychosis: A Meta-Analysis. J Clin Psychiatry. enero de 2017;78(1):e28-40.

13. Reitan RM, Wolfson D. The Halstead-Reitan neuropsychological test battery: theory and clinical interpretation. Tucson, Ariz: Neuropsychology Press; 1985. 486 p.

14. Keefe RSE, Goldberg TE, Harvey PD, Gold JM, Poe MP, Coughenour L. The Brief Assessment of Cognition in Schizophrenia: reliability, sensitivity, and comparison with a standard neurocognitive battery. Schizophr Res. 1 de junio de 2004;68(2-3):283-97.

15. Spreen O, Strauss E. A Compendium of Neuropsychological Tests. New York: xford University; 1991.

16. Thurstone LL. Primary mental abilities. Psychometric Monographs. 1938;1.

17. Wechsler D. Wechsler Abbreviated Scale of Intelligence. San Antonio, TX.: Psychological Corporation; 1999.

18. Golden CJ. Stroop Color and Word Test. Chicago, Illinois: Stoelting Company; 1978.

19. Nuechterlein KH, Edell WS, Norris M, Dawson ME. Attentional Vulnerability Indicators, Thought Disorder, and Negative Symptoms. Schizophr Bull. 1 de enero de 1986;12(3):408-26.

20. Cornblatt BA, Risch NJ, Faris G, Friedman D, Erlenmeyer-Kimling L. The continuous performance test, identical pairs version (CPT-IP): I. new findings about sustained attention in normal families. Psychiatry Res. noviembre de 1988;26(2):223-38.

21. Gold JM. Auditory Working Memory and Wisconsin Card Sorting Test Performance in Schizophrenia. Arch Gen Psychiatry. 1 de febrero de 1997;54(2):159.

22. Petrides M, Alivisatos B, Evans AC. Functional activation of the human ventrolateral frontal cortex during mnemonic retrieval of verbal information. Proc Natl Acad Sci. 20 de junio de 1995;92(13):5803-7.

23. Brandt J, R.H.B. B. The Hopkins Verbal Learning Test—Revised: Professional Manual. Odessa, Fla: Psychological Assessment Resources, Inc.; 2001.

24. Lezak MD. Neuropsychological Assessment. Oxford University Press; 2004.

25. Woods S, Delis D, Scott J, Kramer J, Holdnack J. The California Verbal Learning Test – second edition: Test-retest reliability, practice effects, and reliable change indices for the standard and alternate forms. Arch Clin Neuropsychol. agosto de 2006;21(5):413-20.

26. Delis DC, Kramer JH, Kaplan E, Ober BA. California Verbal Learning Test--Second Edition [Internet]. American Psychological Association; 2016 [citado 4 de octubre de 2022]. Disponible en: http://doi.apa.org/getdoi.cfm?doi=10.1037/t15072-000

27. Wilkinson, G.S.. WRAT-3: Wide Range Achievement Test Administration Manual. Wide Range, Wilmington, DE;1993.

28. Benedict RHB. Benedict RHB: Brief Visuospatial Memory Test—Revised: Professional Manual. Odessa, Fla: Psychological Assessment Resources, Inc.; 1997.

29. Rey A. L’examen clinique en psychologie. Presses Un, Parisi, M.1964.

30. White T, Stern RA. Neuropsychological Assessment Battery: Psychometric and Technical Manual. Lutz, Fla: Psychological Assessment Resources, Inc; 2003.

31. Baron-Cohen S, Jolliffe T, Mortimore C, Robertson M. Another Advanced Test of Theory of Mind: Evidence from Very High Functioning Adults with Autism or Asperger Syndrome. J Child Psychol Psychiatry. octubre de 1997;38(7):813-22.

32. Baron-Cohen S, Wheelwright S, Hill J, Raste Y, Plumb I. The «Reading the Mind in the Eyes» Test revised version: a study with normal adults, and adults with Asperger syndrome or high-functioning autism. J Child Psychol Psychiatry. febrero de 2001;42(2):241-51.

33. Vantwout M, Aleman A, Kessels R, Laroi F, Kahn R. Emotional processing in a non-clinical psychosis-prone sample. Schizophr Res. 1 de junio de 2004;68(2-3):271-81.

34. Corcoran R, Mercer G, Frith CD. Schizophrenia, symptomatology and social inference: Investigating “theory of mind” in people with schizophrenia. Schizophr Res. septiembre de 1995;17(1):5-13.

35. Wechsler D. WAIS-R: Wechsler adult intelligence scale-revised. New York, N.Y.: Psychological Corporation; 1981.

36. Wechsler D. Wechsler Intelligence Scale for Children, 3rd edition New York, N. Y: Psychological Corporation; 1991.

37. Nelson HE. National Adult Reading Test (NART): Test manual. Windsor: NFER-Nelson; 1982.

38. Maurer K, Häfner H. Erste Signale einer Psychose richtig deuten: Checkliste für die Hausarztpraxis in Arbeit. MMW - Fortschritte Med. marzo de 2007;149(13):36-8.

39. Benton, A., Hamsher, K., Sivan, A. Multilingual Aphasia Examination, 3rd ed. AJA Associates, Iowa City, Iowa,1983.

40. Grant DA. Computer version of the Wisconsin Card Sorting Test, WCST. Odessa, Fla: Psychological Assessment Resources; 2000.

41. Stroop JR. Studies of interference in serial verbal reactions. J Exp Psychol. diciembre de 1935;18(6):643-62.

42. Finger Tapping Test [computer program]. Lelystad: BuroTesteR; 2002.

43. Doty RL, Shaman P, Kimmelman CP, Dann MS. University of pennsylvania smell identification test: A rapid quantitative olfactory function test for the clinic. The Laryngoscope. febrero de 1984;94(2):176-8.
